# Dr. Evelyn Baldwin

**Published:** 1917-05

**Authors:** 


### OBITUARY

**Readers are requested to report promptly the death of all physicians in Western New York, or former residents of this region, or graduates of any medical school in Western New York, and to notify the families of the deceased of our desire to publish adequate Obituary notices.**

**Dr. Evelyn Baldwin,** Women's Medical College of N. Y., 1892, died at her home in Rochester March 24, of pneumonia following grippe, aged 55.

